# Utilization of non-pharmacological methods and the perceived barriers for adult postoperative pain management by the nurses at selected National Hospitals in Asmara, Eritrea

**DOI:** 10.1186/s12912-020-00492-0

**Published:** 2020-10-22

**Authors:** Betiel Yihdego Kidanemariam, Traudl Elsholz, Laban L. Simel, Eyasu H. Tesfamariam, Yonatan Mehari Andemeskel

**Affiliations:** 1 Department of Anesthesia and Critical Care, School of Nursing, Asmara College of Health Sciences, Asmara, Eritrea; 2 School of Public Health, Asmara College of Health Sciences, Asmara, Eritrea; 3 Department of Epidemiology and Biostatistics, School of Public Health, Asmara College of Health Sciences, Asmara, Eritrea

**Keywords:** Non-pharmacological pain management methods, Pain management, Perceived barriers, Postoperative pain

## Abstract

**Background:**

Pharmacological methods are widely used for postoperative pain management however, poorly controlled pain continues to pose a significant challenge. Non pharmacological methods could contribute to the unresolved postoperative pain management in assisting nurses’ routine care and reducing the need for medication. This study aimed to assess nurses’ utilization of non-pharmacological methods in postoperative pain and the perceived barriers for their implementation at the National Hospitals.

**Methods:**

This was a descriptive cross sectional study conducted among 154 nurses working at the National Referral Hospitals and Sembel Private Hospital. A standardized five-point Likert-scale questionnaire which assesses nurses’ utilization of selected non-pharmacological methods and the perceived barriers for the implementation was used to collect data. Descriptive statistics for the demographic data, independent samples t-test, one way ANOVA and factorial ANOVA were used to analyze the data. Statistical significance level was set at *P < 0.05*.

**Results:**

The study found out that emotional support (45.5%), helping with daily activities (67.5%) and creating a comfortable environment (61%) were mostly used while, cognitive-behavioral (5.9%) and physical methods (5.8%) were hardly used. The results also showed that, characteristics such as, age (*p* = 0.013), level of education (*p* = 0.012), work experience (*p* = 0.001) and place of work (*p* = 0.001), were significantly related to the use of non-pharmacological methods at bivariate level. However, hospitals were the only determinants of the non-pharmacological methods at multivariable level with a statistical significance of (*p* < 0.001). On the perceived barriers; heavy work load (87.7%), shortage of time (84.4%), limited resources (82.5%), deficit in the guidelines for pain management (77.3%), patient’s uncooperative behavior (57.1%), language difference (64.4%), nurse’s lack of knowledge (50%) and experience (40.3%) were identified.

**Conclusion:**

The use of non-pharmacological methods in the studied hospitals varied greatly due to knowledge and experience of the nurses. Therefore, it is recommended that exposure and training for all health care providers at all level is a paramount importance in order to appreciate the benefits of non-pharmacological methods applicable to postoperative pain management. This could be achieved through on job training, seminars, scientific conferences and other brainstorming forums.

**Supplementary information:**

**Supplementary information** accompanies this paper at 10.1186/s12912-020-00492-0.

## Background

Postoperative pain is the most common problem in developing and developed countries with reported incidence ranging between 47 and 100% [1]. Despite its longstanding recognition and many advances in the management, poorly controlled postoperative pain continues to pose a significant challenge [2–5]. Unmanaged postoperative pain can predispose patients to medical complications like pneumonia, deep vein thrombosis, delayed wound healing, chronic pain and increased hospital stay which may result in patients dissatisfaction [6, 7]. The cornerstone of effective postoperative pain management is based on the nurses’ ability to assess pain [8]. The effective administration and use of pain relievers, depends on the surgical nurse’s knowledge and skills to assess postoperative pain [5]. The main goal of postoperative pain treatment is to comfort and facilitate healing to patients after surgical procedures by inhibiting nociceptive impulses induced by trauma [9].

Pharmacological methods such as, opioids and NSAID are often used in alleviating post-operative pain [8]. The above methods have potential risks and high cost, as patients may develop drug dependency, severe adverse effects such as respiratory depression which is life threatening and vital organ damage. Inconsistency in the procurement of opioids hinders their availability in developing countries [10]. In the Eritrean settings, frequent administration of pharmacological methods in postoperative care could expose patients to these risks.

Non-pharmacological methods refers to diverse approaches that involve nondrug measures to relief pain [11], which includes deep breathing, exercises, massage, positioning and music therapy. According to Pölkki et al., [12] non-pharmacological methods are divided into five categories cognitive-behavioural methods, physical methods, emotional support, helping with activities of daily living and creating a comfortable environment. These interventions are recognized as valuable, simple, safe, inexpensive with less adverse effects and promote active role of patients in the management of pain [13]. They can be used independently for the relief of mild pain or offer complementary management options in conjunction with pain medication for the relief of moderate to severe pain [14]. Non pharmacological methods could assist on the nurses’ routine care and reduce the need for higher dosing of medications [15]. Unlike in pharmacological methods, nurses have full authority on the implementation of non-pharmacological methods in postoperative pain management. Medicinal drugs are widely used in the management of somatic pain, while non-pharmacological therapies aim to treat the affective, cognitive, behavioral and socio-cultural types of pain [8, 16]. Utilization of non-pharmacological methods by the nurses could contribute to the unresolved postoperative pain resulting from the administration of pharmacological methods only [17]. The overwhelming safety and benefits associated with non-pharmacological methods are usually overlooked and less utilized [18]. Several barriers were identified, such as knowledge and experience of the nurses, inadequate time, heavy workload, shortage of nurses, lack of patients’ participation,failure in administrative support and resource scarcity [17, 19–21].

In the post-operative surgical wards of the studied hospitals in Asmara, the use of pharmacological methods is the primary approach of pain management however, postoperative pain remains as a challenge with high level of patient’s dissatisfaction [22, 23] Therefore, the aim of the study was to assess the utilization of the non-pharmacological methods and the perceived barriers which limit their utilization in the selected sites.

## Methods

### Study design and study setting

This was a descriptive cross-sectional study design conducted in the period between February and March of 2018.A quantitative approach was used to assess the utilization of the non-pharmacological methods and perceived barriers among the nurses. The study was carried out in Halibet National Referral Hospital (HNRH), Orotta National Referral Hospital (ONRH) and Sembel Private Hospital (SPH) in Asmara, Eritrea.

### Study participants

**Fig. 1:**
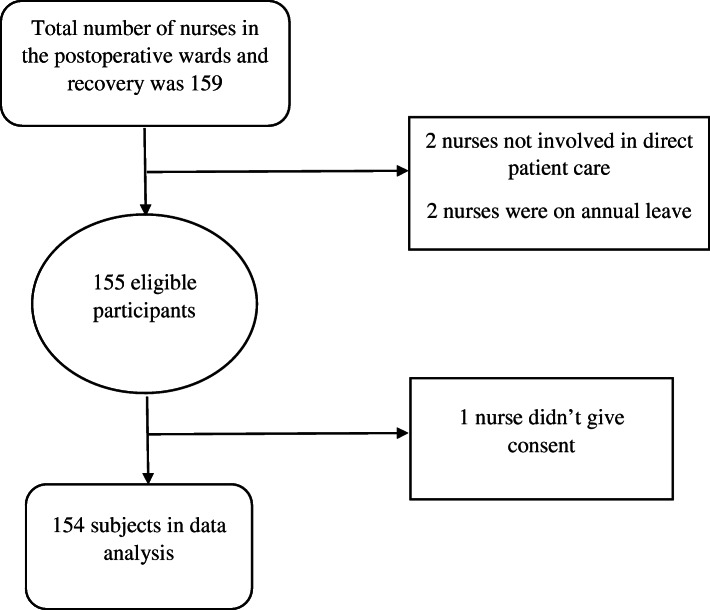
Eligible number of patients for the study

Out of 159 number of nurses in the postoperative surgical wards and recovery units in the above mentioned hospitals, 154 nurses met the inclusion criteria. Nurses of all age group, gender, various level of education, currently working in post-operative and recovery units of the three selected hospitals were among the studied group (Fig. 1).

### Questionnaire

A structured questionnaire developed by Pölkki et al. [12] was used in this study after a formal permission was obtained. The questionnaire had previously been adopted by studies done in China and Singapore [17–19]. The questionnaire was further modified to fit the study area and study population. For example educational level in the Eritrean curriculum categorizes nurse’s education into three levels; ‘associate nurse’, ‘diploma nurse’ and ‘degree nurse’. The questionnaire had three sections, the first section comprised the key elements of socio-demographic and work experience data which included (8 entities). The second section of the questionnaire was about nurses’ use of non-pharmacological methods in pain management. The non-pharmacological methods were divided into 5 categories; cognitive-behavioral methods (6 entities) in which preparatory information had (18 entities), physical methods (4 entities), emotional support (3 entities), helping in daily activities (1entity) and creating a comfortable environment (1entity). The third section inquired the perceived barriers (14 entities) that hinder nurse’s implementation on the non-pharmacological methods.

### Data collection procedure

Permission for conducting the research was initially obtained from the ethical committee of Asmara College of Health Sciences, Ministry of Health at the department of research and human resource development. Further permission was obtained from the study sites in this case, the hospitals after an organized meeting with the hospital heads prior to carrying out the study. Verbal and written informed consent was obtained from the participants before administering the questionnaire. A researcher administered structured questionnaire was used for data collection. All researchers involved in this study undertook 2 days of training on how to administer the questionnaire during data collection. Data was collected in the morning and evening shifts whereby convenient time for the participants was during half an hour break of their work schedule. To avoid errors in the data collection, researchers at certain occasions translated the questions to the local language. Each of the questionnaire was promptly checked for a range of valid values and completion of all items without any skip, before leaving from each data collection site.

### Variable measurement

The five components on nurse’s utilization of non-pharmacological methods were ‘cognitive behavioral methods’, ‘physical methods’, ‘emotional support’, ‘helping with daily activities’ and ‘creating a comfortable environment’. Questions in this section were standardized in five point Likert scale: ‘not at all’, ‘very seldom’, ‘sometimes’, ‘nearly always’ and ‘always’. The section that measured the perceived barriers that hinder nurse’s implementation was standardized to five point Likert response: ‘strongly agree’, ‘agree’, ‘neither agree nor disagree’, ‘disagree’ and ‘strongly disagree’.

### Validity and reliability

The validity and internal consistency in the previous findings suggested that the overall questionnaire had adequate validity and reliability [17, 19]. An expert panel consisting of an anesthesiologist, a professor in critical care nursing and a senior surgical nurse was organized to evaluate face and content validity of the questionnaire. The reliability analysis in this study also indicated that the scales had good internal consistency. The cronbach’s alpha for preparatory information (18entities), non-pharmacological methods (15entities) were 0.89, 0.87respectively.

Factor structure was observed using exploratory factor analysis for features that hinder nurses from performing non-pharmacological methods. Three factors were identified using principal component analysis with varimax rotation. The KMO statistic was 0.69 and significant (*p* < 0.001) Bartlett’s sphericity was found. The cronbach’s alpha for the perceived barriers was 0.75.

### Data analysis

Data was cleaned, coded and entered into SPSS (version 22) for analysis. Internal consistency of the items were checked using Cronbach α before conducting the main analysis. Frequency, percentage and median (IQR) were used for descriptive analysis. To simplify presentation, the items of non-pharmacological methods collected in five-point Likert scale were condensed into three, by merging the adjacent responses (Nearly always/always, sometimes, not at all/seldom). Comparison across the different categories of the nurses’ demographic characteristics and work experiences regarding their utilization of the non-pharmacological methods were performed using independent samples t-test and one way ANOVA (accompanied with LSD post hoc analysis for the significant variables). Variables that were significant at bivariate level were selected for further analyses at multivariable level using factorial ANOVA. *P*-values less than 0.05 were considered as significant in all the analyses.

## Results

### Nurses socio-demographic characteristics

The majority of the study participants were females whose number was74%.The age of the participants ranged between 20 and 70 years with a median age of 27 years. A good proportion of the nurses (53.2%) were certificate holders with 1 year training. Among the nurses 38.3% had 3 to 5 years of experience of which 48.1% of them had 2 years of surgical care experience. About 57.1% of the participants were from Orotta hospital. The rest of the demographic and clinical details of the participants are shown in Table 1.

**Table 1:** Socio-demographic characteristics of Nurses (*N* = 154)

Characteristics		Frequency(n)	Percent (%)
**Gender**	Male	40	26.0
	Female	114	74.0
**Age, years** (Md = 27, IQR = 6, Min. = 20 and Max. = 70)	20 to 36	126	81.8
	37 to 53	22	14.3
	54 to 70	6	3.9
**Experience in Health care (years)** (Md = 5, IQR = 5, Min. = 1 and Max. = 45)	0 to 2	30	19.5
	3 to 5	59	38.3
	6 to 10	37	24.0
	≥11	28	18.2
**Experience in surgical (years)** (Md = 3, IQR = 4, Min = 1 and Max = 26)	0 to 2	74	48.1
	3 to 5	51	33.1
	6 to 10	20	13.0
	≥11	9	5.8
**Educational status**	Associate Nurse	82	53.2
	Diploma Nurse	67	43.5
	Degree Nurse	5	3.3
**Hospital**	HNRH	36	23.4
	ONRH	88	57.1
	SPH	30	19.5
**Multi professional cooperation**	Good	127	82.5
	Moderate	25	16.2
	Poor	2	1.3
**Hospitalized Close relative**	Yes	34	22.1
	No	120	77.9

*Md* Median, *IQR* Interquartile range, *Min.* Minimum, *Max.* Maximum, *HNRH* Halibet National Referral Hospital, *ONRH* Orotta National Referral Hospital, *SPH* Sembel Private Hospital

### Nurses utilization on non-pharmacological methods for post-operative pain relief

The summary on the utilization of non-pharmacological methods is illustrated in Table 2. The cognitive behavioral methods, breathing (81.7%), and relaxation (72.1%) techniques were mostly reported to be utilized as ‘*nearly always/always*’ where as positive reinforcement (79.3%), distraction (77.5%), and imagery (70.1%) methods were utilized as ‘*not at all/seldom*’.

**Table 2:** Nurses’ utilization of non-pharmacological methods

Non pharmacological Methods	Not at all/ Seldom (%)	Sometimes (%)	Nearly always/ Always (%)
**Cognitive-behavioural methods**			
Positive re-inforcement	79.3	20.7	0
Breathing Technique	3.9	14.4	81.7
Relaxation	3.2	24.7	72.1
Distraction	77.5	18.6	3.9
Imagery	70.1	20.2	9.7
Preparatory Information^a^	12.3	65.0	22.7
**Physical methods**			
Heat Thermal Regulation	76.0	12.3	11.7
Cold Thermal Regulation	72.7	17.5	9.7
Massage	50.0	31.2	18.8
Positioning	6.5	9.1	84.4
**Emotional**			
Presence	17.5	37.0	45.5
Comforting and reassurance	0.6	7.1	92.2
Therapeutic touch	32.5	28.5	39.0
**Helping with daily activities**	7.8	24.7	67.5
**Creating a comfortable environment**	15.5	39.0	45.5

^a^ Preparatory information inclusive of cognitive, sensory and ways of giving information

Among the physical methods, alleviating pain by positioning the patient was responded by most (84.4%) of the nurses as ‘*nearly always/always*’ whereas thermal regulation was the least used. In emotional support, therapeutic touch was reported as *‘not at all/seldom’* by (32.5%) of the nurses, while comfort and reassurance were used more often at 92.2%.

Concerning preparatory information (Table 3) on the cognitive information preparation for procedure and pain medication after the procedure’ were the most commonly addressed items as ‘*nearly always/always*’ by (95.2%) and (89.8%)of the nurses. Informing patients on the length of the procedure was at 54.4% whereas, the type of anesthesia was at 78.9% being used *‘not at all/ seldom’*. On ways of giving information regarding sensation majority of the nurses (85.7%) responded as ‘*nearly always/always’*.

**Table 3:** Item wise response of nurses on preparatory information as a method of pain management (*N* = 154)

Preparatory Information	Not at all/ Seldom (%)	Sometimes (%)	Nearly always/ Always (%)
***(1) Cognitive and Sensory Information***			
**Cognitive Information**	12.2	10.2	77.6
Types of procedure			
Place of procedure	14.3	6.1	79.6
Person who carries out procedure	35.4	8.8	55.8
Purpose of procedure	23.8	20.4	55.8
Duration of the procedure	54.4	27.9	17.7
Preparation of procedure	3.4	1.4	95.2
Type of anesthesia	78.9	10.9	10.2
Postoperative placement	29.3	15.6	55.1
Postoperative monitoring’s	21.8	12.9	65.3
Patients limitation	7.1	5.2	83.1
Pain medication after procedure	6.5	3.2	85.7
**Sensory Information**			
Preoperational sensations (eg. Fear, anxiety)	48.7	22.1	29.2
Sensation during the procedure	77.3	13.6	9.1
Post operational sensation	26.0	11.7	62.3
***(2) Ways of giving information***			
Encouraging the patient to ask about misconception	10.4	27.3	62.3
Talking openly about sensation	4.5	9.7	85.7
Giving information honestly and openly	5.2	4.5	90.3
Making sure that the information has been understood	11.7	35.1	53.2

### Utilization of non-pharmacological methods and nurses’ background factors

Significant differences (Table 4) in utilization of non-pharmacological methods were found across the categories of age groups (*p* = 0.013), educational level (*p* = 0.012), hospital (*p* = 0.009), experience in health care (*p* = 0.001), and prior experience of hospitalization of a close relative (*p* = 0.008).Statistically significant difference were also found among nurses providing preparatory information across their educational level (*p* = 0.015), place of work (*p* < 0.001), experience in health care (*p* = 0.002), availability of pain assessment tools (*p* < 0.001) and prior experience of hospitalization (*p* = 0.004).

**Table 4:** Comparison on utilization of non-pharmacological methods across categories of background characteristics of nurses (*N* = 154)

Variable		Mean (SD)	p-value	p-trend
**Age**	20 to 24	105.7 (20.0)	**0.013**	0.004
	25 to 29	105.4 (17.3)		
	30 to 39	110.9 (16.5)		
	40 or above	118.2 (15.5)		
**Educational status**	Associate Nurse	104.5 (19.0)	**0.012**	0.003
	Diploma Nurse	111.5 (16.5)		
	Degree Nurse	122.8 (14.9)		
**Hospital**	Halibet	110.1 (19.5)	**0.009**	0.001
	Orotta	101.4 (15.7)		
	Sembel	125.7 (10.0)		
**Experience in Health Care, years**	0 to 2	102 (21.8)	**0.001**	0.006
	3 to 5	109.3 (17.4)		
	6 to 10	103.5 (16.0)		
	11 to 45	118.7 (13.9)		
**Experience in surgical, years**	0 to 2	107 (19.5)	0.196	0.181
	3 to 5	106 (17.0)		
	6 to 10	111 (17.6)		
	11 to 26	119.5 (13.0)		
**Availability of pain assessment tool**	No	106.8 (17.6)	0.050	–
	Yes	134 (8.4)		
**Hospitalization with a close relative**	No	106 (18.0)	**0.008**	–
	Yes	115.5 (17.3)		

LSD post hoc analysis (Additional file 1) revealed that nurses older than 40 years had significantly higher utilization compared to 20–24 years (*p* = 0.005), and 25–29 years (*p* = 0.002). In addition, nurses at associate level had significantly less utilization compared to those at diploma (*p* = 0.010) and degree (*p* = 0.020) level.

On multivariable analysis (Table 5), ‘hospital’ was the only determinant in the utilization of non-pharmacological methods (*p* < 0.001).

**Table 5:** Factorial analysis of overall non-pharmacological methods (*N* = 154)

Variables	***p***-value	Partial Eta Squared	Observed Power
Educational level	0.414	0.012	0.201
Hospital	**< 0.001**	0.246	1.000
Age	0.618	0.012	0.172
Experience in Health care	0.236	0.029	0.373

### Perceived barriers for the utilization of non-pharmacological methods for postoperative pain management

Among the wide range of related perceived barriers in the utilization of non-pharmacological methods, lack of administrative support was the least (76.6%) among the nurses as health care system-related barrier, while lack of knowledge (50.0%)was highly rated as important nurse related barriers. A considerable number of the nurses (64.9%) indicated that the language difference was relevant as patient related barrier Table 6.

**Table 6:** Perceived barriers for the utilization on non-pharmacological methods (*N* = 154)

Barriers	Agree n (%)	Neutral n(%)	Disagree n(%)
**Healthcare System-Related Barriers**			
Heavy work load	135 (87.7)	6 (3.9)	13 (8.4)
Lack of time	130 (84.4)	5 (3.2)	19 (12.3)
Lack of administrative support	118 (76.6)	3 (1.9)	33 (21.4)
Lack of resources (e.g. equipment’s, materials)	127 (82.5)	5 (3.2)	22 (14.3)
Lack of pain management policy	119 (77.3)	1 (0.6)	34 (22.1)
**Nurses-Related Barriers**			
Personal interest	26 (16.9)	8 (5.2)	119 (77.3)
Lack of knowledge regarding non-pharmacological pain relief methods	77 (50)	4 (2.6)	73 (47.4)
Lack of experience in using non-pharmacological methods	62 (40.3)	5 (3.2)	87 (56.5)
Personal, traditional and cultural values on pain and pain relief methods	45 (29.4)	9 (5.9)	99 (64.7)
Belief that other health team members should take main role	20 (13)	7 (4.5)	127 (82.5)
Belief nurses primary task is to administer pain medication for pain relief	43 (27.9)	3 (1.9)	108 (70.1)
Belief inefficacy of non-pharmacological methods in pain relief	49 (31.8)	11 (7.1)	94 (61.0)
**Patient-Related Barriers**			
Patients inability to cooperate	88 (57.1)	20 (13)	46 (29.9)
Language difference	100 (64.9)	18 (11.7)	36 (23.4)

## Discussion

This study discusses the utilization of the non-pharmacological methods for alleviating postoperative pain and existing barriers. The study was conducted using a standard questionnaire which was applied in different settings, making it useful for comparisons. Since the study was conducted in the major hospitals, it gave an informative picture of the degree of utilization of these methods.

### Utilization of non-pharmacological methods for postoperative pain management

This study found out that non-pharmacological pain reliving methods are less utilized by the nurses. A recent study conducted among Eritrean nurses indicated a gap in the knowledge and attitude in pain management. This knowledge gap seems to have resulted from inadequate training in the nursing curriculums [22]. A study conducted in the same setting reported negligence in pain management [23]. Moreover, the common complains in the National hospitals have been shortage in number of nurses which hindered the utilization of these methods.

Among the cognitive behavioral methods, breathing and relaxation were often used, while the rest of the methods were the least used. In support to the above assertion, a study done by Faigeles et al., [24], stated that the most frequently used non-pharmacological interventions during acute pain were calming voice, providing information, and deep breathing. It had been rationalized that those methods are easy to implement and doesn’t require equipment or specific training [24]. A study done in Westminister, England on non-pharmacologic pain management for postoperative coronary artery bypass in graft surgical patients, reported that deep breathing followed by distraction were commonly used as pain relieving methods. The majority of the patients stated it helped them to minimize both the perception and sensation of pain in contrast to pharmacological methods alone [25].

The preparatory information involved entities done at the preoperative period and in this current study, they were found to be the least used. This was similar to a study conducted in Singapore [19], in which items; sensory information, paying enough attention to the ways of giving information, and the use of materials to help with informing were found to be less frequently utilized. In both studies those findings could be attributed to the fact that preparatory information is provided mainly by surgeons and/or anesthetists.

Alleviating pain by positioning of patients was the most frequently used physical method due to its routine application, acceptability, easy to administer and requires less time compared to the other methods. Massage application and thermal regulation with heat and cold application were rarely used. This is explained by lack of heating and cooling devices, lack of training and cultural influences. Similar studies done in Finland, China and Singapore reported that positioning was the most commonly used physical method during postoperative pain, whereas, thermal regulation and massage were the least used [12, 17, 19]. Furthermore, Kooten [25] stated that positioning was frequently used, whereas cold packs were rarely used. Another study conducted in Finland on postoperative pain management after hip surgery, stated that positioning was the most commonly used non-pharmacological method [26]. A study done by Gelinas and his colleagues, nurses were found to practice simple massaging as a pain relieve method on critically ill patients [27].

In this study on emotional support, nurses presence was (45.5%) which was less frequently utilized compared to the Finland (77%) and Singapore (49%) nurses on relieving postoperative pain [12, 19]. This discrepancy could be explained by the less time surgical nurses spend with patients or nurses lack of understanding on the actual meaning of presence. In this study therapeutic touch was less used by the nurses. Results of Bonnie Faigeles [24], was incongruent with this study, stating that nurses provided pain relief by frequently holding hands and gentle touch during acute pain. This study found out that the nurses always helped patients during their daily activities and created a comfortable environment. This finding concurred with a study done by He et al., [19] which reported similar findings.

Over all nurses’ age, educational status, experience in healthcare and prior hospitalization with a close relative had a significant difference in the utilization of non-pharmacological methods. The utilization of non-pharmacological methods is proportionate to the increase in age, experience and educational level of the nurses. These findings were similar to a study carried out in Finland, China and Singapore which found out nurses age, educational status, work of experience had effects on the use of some non-pharmacological methods [12, 17, 19].

Statistical differences prevailed across the provision of preparatory information, the nurses’ educational level, work place, experience in health care, availability of pain assessment tools, and prior experience of hospitalization. This study findings was congruent to the research study done by Pölkki in 2001 where by *‘the nurses’ age, education and nursing experience showed statistically significant associations with the preparatory information.’* There was a statistical significance among the study sites in which variation on the use of the non-pharmacological methods was disproportionately distributed. The uneven distribution resulted from the type of services, their specialties, affordability of the services, the catchment population served, accessibility and previous experience.

### Perceived barriers that hinder the utilization of non-pharmacological methods

A number of barriers on the utilization of the non-pharmacological methods has been perceived by the nurses and the barriers were related to health care system, nurses and patients related indicating a statistical significance of *(p* < 0.001).

In this study health care system related barriers such as heavy workload, less time and limited equipment’s were the most commonly perceived barriers. This study was consistent with the study done in 2011, in which heavy work load and less time were reported as barriers [28]. A study done by Batiha in 2014 identified lack of pain management policies, lack of proper pain assessment tool, less number of nurses, interruptions of activities relating to pain and unavailability of alternative non-pharmacologic therapy as healthcare related barriers [29]. A similar study done in Poland, found out that limitations such as lack of standard operating procedures and guidelines by the administration interfered with pain management [18]. Moreover, a study done in China stated the most common limiting factors were less nurse to patient ratios and heavy workload leading to less attention on post-surgical pain which was consistent with the findings of this study [17].

On nurse related barriers, lack of knowledge and experience on non-pharmacological methods were stated as existing barriers. This was associated with less experience and specialization on non-pharmacological methods and poor positive opinion on its efficiency. This study finding agreed with the results of Batiha in 2014 in which time limitations, less communication and inadequate staff knowledge were pointed out. In addition to this, a study done in South Korea indicated that time constraints and insufficient knowledge were the most encountered barriers [30]. Furthermore, a study done in Iran identified limited nurse-patient interaction, lack of pain management interventions and inadequate time to deliver non-pharmacological pain relief measures as barriers [31]. On patient related barriers lack of cooperation and the limitation in language were found out to affect negatively the use of non-pharmacological methods in pain management. In this study it is postulated that positive attribute to the pharmacological methods hinders the effective use of the non-pharmacological methods.

### Study limitations

The use of questionnaires in data collection for this study might have created limitation on the side of respondents since, selected non-pharmacological methods were captured. This study took in to account non-pharmacological methods as practiced at major referral facilities hence, the picture could be different if the study was all inclusive of other lower level facilities. The study did not focus on the benefits attributed to the combination of non-pharmacological methods and pharmacological methods.

## Conclusion

This study concluded that the nurse’s use of the non-pharmacological methods on postoperative patients in the studied hospitals varied greatly due to knowledge and experience. The study recommends exposure and training for all health care providers at all levels in order to appreciate the benefits of non-pharmacological methods applicable to postoperative pain management among patients. This could be achieved through journal publications, on job training, seminars, scientific conferences and other brainstorming forums.

## Supplementary information

**Additional file 1.** Post-hoc analysis results.

## Data Availability

The complete dataset supporting the conclusions of this article is available from the corresponding author and can be accessed upon a reasonable request.

## Abbreviations

ANOVA: Analysis of Variance
NT: Ear Nose and Throat
IQR: Interquartile Range
KMO: Kaiser-Meyer-Olkin
LSD: Least Significant Difference
NSAID: Non-steroidal Anti-inflammatory Drugs
