# Supplementary material for: Utilization of non-pharmacological methods and the perceived barriers for adult postoperative pain management by the nurses at selected National Hospitals in Asmara, Eritrea
Source: BMC Nurs. 2020 Oct 22;19:100. doi: 10.1186/s12912-020-00492-0 (PMC7583254; doi:10.1186/s12912-020-00492-0)
Supplement: Supplementary file 1 — Additional file 1. Post-hoc analysis results. [file 12912_2020_492_MOESM1_ESM.pdf]

Additional file 1

Table: Post hoc analysis of overall non-pharmacological methods (N=154)

Variables			Mean diff.	p-value
**Age**				
	20-24	25-29	0.32	0.900
		30-39	-5.22	0.200
		>40	-12.9	**0.005**
	25-29	30-39	-5.5	0.243
		>40	-13.2	**0.002**
	30-39	>40	-7.6	0.123
**Educational status**				
	Associate	Diploma	-7	**0.012**
		Degree	-18.2	**0.021**
	Diploma	Degree	-11.2	0.131
**Hospital**				
	HNRH	ONRH	8.6	**0.006**
		SPH	-15.5	**<0.001**
	ONRH	SPH	-24.2	**<0.001**
**Experience in health care, years**				
	0 to2	3 to 5	-7.2	0.060
		6 to10	-1.4	0.132
		>11	-16.6	**0.025**
	3 to 5	6 to 10	5.8	0.165
		>11	-9.3	**0.001**
	6 to 10	>11	-15.2	**0.001**
